# GASTRIC NEUROENDOCRINE TUMOR: WHEN SURGICAL TREATMENT IS INDICATED?

**DOI:** 10.1590/0102-672020230050e1768

**Published:** 2023-10-13

**Authors:** Ademar Caetano Assis, Valdir Tercioti, Nelson Adami Andreollo, José Antonio Possatto Ferrer, João de Souza Coelho, Luiz Roberto Lopes

**Affiliations:** 1 Universidade Federal de Jataí – Jataí (GO), Brazil; 2 Universidade Estadual de Campinas, Faculty of Medical Sciences, Department of Surgery and Gastro Center – Campinas (SP), Brazil

**Keywords:** Stomach neoplasms, Neuroendocrine tumors, Surgical oncology, Neoplasias gástricas, Tumores neuroendócrinos, Oncologia cirúrgica

## Abstract

**BACKGROUND:**

Gastric neuroendocrine tumors are a heterogeneous group of neoplasms that produce bioactive substances. Their treatment varies according to staging and classification, using endoscopic techniques, open surgery, chemotherapy, radiotherapy, and drugs analogous to somatostatin.

**AIMS:**

To identify and review cases of gastric neuroendocrine neoplasia submitted to surgical treatment.

**METHODS:**

Review of surgically treated patients from 1983 to 2018.

**RESULTS:**

Fifteen patients were included, predominantly female (73.33%), with a mean age of 55.93 years. The most common symptom was epigastric pain (93.3%), and the mean time of symptom onset was 10.07 months. The preoperative upper digestive endoscopy (UDE) indicated a predominance of cases with 0 to 1 lesion (60%), sizing ≥1.5 cm (40%), located in the gastric antrum (53.33%), with ulceration (60%), and Borrmann III (33.33%) classification. The assessment of the surgical specimen indicated a predominance of invasive neuroendocrine tumors (60%), with angiolymphatic invasion in most cases (80%). Immunohistochemistry for chromogranin A was positive in 60% of cases and for synaptophysin in 66.7%, with a predominant Ki-67 index between 0 and 2%. Metastasis was observed in 20% of patients. The surgical procedure most performed was subtotal gastrectomy with Roux-en-Y reconstruction (53.3%). Tumor recurrence occurred in 20% of cases and a new treatment was required in 26.67%.

**CONCLUSIONS:**

Gastric neuroendocrine tumors have a low incidence in the general population, and surgical treatment is indicated for advanced lesions. The study of its management gains importance in view of the specificities of each case and the need for adequate conduct to prevent recurrences and complications.

## RESUMO

**RACIONAL:**

Os tumores neuroendócrinos gástricos são um grupo heterogêneo de neoplasias produtoras de substâncias bioativas, sendo o seu tratamento variável de acordo com o estadiamento e a classificação, sendo utilizadas técnicas endoscópicas, cirurgias abertas, quimioterapia, radioterapia e fármacos análogos da somatostatina.

**OBJETIVOS:**

Identificar e revisar os casos de neoplasia neuroendócrina gástrica submetidos a tratamento cirúrgico.

**MÉTODOS:**

Revisão os doentes tratados cirurgicamente de 1983 e 2018.

**RESULTADOS:**

Foram incluídos 15 pacientes, com predomínio do sexo feminino (73,33%) e média de idade de 55,93 anos. O sintoma mais comum foi a epigastralgia (93,3%) e o tempo médio do início dos sintomas foi de 10,07 meses. A endoscopia digestiva pré-operatória indicou predomínio de casos com 0 a 1 lesões (60%), de tamanho ≥ 1,5 cm (40%), localizadas em antro gástrico (53,33%), com ulceração (60%), Borrmann 3 (33,33%). A avaliação da peça cirúrgica indicou um predomínio de tumores neuroendócrinos invasivos (60%), com invasão angiolinfática na maioria dos casos (80%). A imuno-histoquímica para cromogranina A foi positiva em 60% dos casos e para sinaptofisina em 66,7%, com índice de Ki-67 predominante entre 0 e 2%. Metástases foram observadas em 20% dos casos. O procedimento cirúrgico mais utilizado foi a gastrectomia subtotal com reconstrução em Y de Roux (53,3%). Recidiva tumoral ocorreu em 20% dos casos e novo tratamento foi necessário em 26,67% dos casos.

**CONCLUSÕES:**

Os tumores neuroendócrinos gástricos apresentam baixa incidência na população em geral, e o tratamento cirúrgico está indicado nas lesões avançadas. O estudo de seu manejo ganha importância frente às especificidades de cada caso e a necessidade de conduta adequada para a prevenção de recidivas e complicações.

## INTRODUCTION

Neuroendocrine tumors (NET) are uncommon neoplasms and represent about 0.5% of new cancer cases^37^. Its incidence ranges from 3.7 to 30%^17^, and the stomach is the most common site of all NETs^30^. The incidence rates of gastric NETs as well as other NETs are on the rise, possibly due to the great detection in endoscopic and histopathological exams^13,36^.

Regarding histomorphological characteristics, gastric NETs can be classified into four main subgroups^26^:

Type I: It is the main type of neuroendocrine tumor, responsible for about 70–80% of cases and usually associated with chronic atrophic gastritis. It presents as multiple tumors, ranging from 1 to 2 cm, and with low metastatic potential^22,26^.

Type II: Tumors are histologically similar to type I but differ in that they are associated with Zollinger-Ellison syndrome or multiple endocrine neoplasia syndromes type 1 (MEN-1). These are rare tumors that, like type I, present as multiple small nodules. The metastatic potential is still low, but it is more significant than type I tumors^10,14,26^.

Type III: They are sporadic, single, large lesions with a high infiltrative and metastatic potential, especially in cases of poorly differentiated tumors. Usually, preexisting pathological conditions are not observed, as occurs in type I and II^26,33^.

Type IV: They are rare and present as single, large, poorly differentiated lesions with high infiltrative and metastatic potential. In addition to the characteristics already described, type IV tumors differ from the other subtypes, because they are not associated with cells similar to the enterochromaffin cells. They derive from other endocrine cells, which secrete hormones such as serotonin, gastrin and adrenocorticotropic hormone^26,35^.

In 2017, the WHO proposed a new classification of gastrointestinal NETs. They were divided into well-differentiated neuroendocrine neoplasms, subdivided into G1 neuroendocrine tumor (mitotic index <2%) and G2 (mitotic index between 2 and 20%), and poorly-differentiated neuroendocrine neoplasms, which include the G3 neuroendocrine carcinoma (mitotic index >20%), subdivided into small cell, large cell, and mixed neoplasms^28,38^.

The definitive diagnosis of the lesions is by biopsy, usually performed during upper digestive endoscopy (UDE). It is recommended an immunohistochemical analysis, including analysis of chromogranin A and synaptophysin for diagnosis, and the Ki-67 proliferative index, indicative of prognosis^21,32,36,39^.

Currently, most cases of gastric NET are treated endoscopically. Surgery is reserved for those in which endoscopic resection cannot be performed or that present poor prognosis factors, such as deep invasion and lymph node metastases^4,19,36^.

The objective of the present study was to identify and review the cases of gastric neuroendocrine neoplasia that underwent surgical treatment.

## METHODS

The medical records of patients with gastric NETs diagnosed between 1983 and 2018 and treated surgically were reviewed. The study was approved by the Ethics Committee of the State University of Campinas (Unicamp), under CAAE 78447517.0.0000.5404.

## RESULTS

The study included 15 patients, of which four (26.67%) were male and 11 (73.33%) were female. Age ranged from 20 to 77 years, with an average of 55.93 years.

Computed tomography (CT) was performed on 14 patients (93.33%), and liver metastasis was found in three of them (20%). Octreoscan was performed in four cases (26.67%), with liver and bone metastasis detected in one patient (6.67%) and liver and lymph node metastasis in another (6.67%).

Comorbidities and other concomitant diseases are described in Table 1. Patients with arterial hypertension and other cardiovascular diseases predominated (53.33%). Previous smoking was reported in six patients (40%) and alcohol consumption in five (33.33%).

**Table 1:** Clinical characteristics of the 15 patients studied.

Comorbidities		n (%)
	High blood pressure and cardiovascular disease*	8 (53.33)
	Thyroid diseases†	4 (26.67)
	Diabetes mellitus	2 (13.33)
	Gastrointestinal diseases‡	5 (33.33)
	Neoplasia§	2 (13.33)
	Others diseases//	7 (46.67)
	Smoking	6 (40.00)
	Alcoholism	5 (33.33)
	Previous surgeries¶	3 (20.00)
**Clinical symptoms**		
	High digestive bleeding	5 (33.33)
	Gastric stasis	7 (46.67)
	Vomits	4 (26.67)
	Anemia	1 (6.67)
	Weight lost	7 (46.67)
	Anorexia	3 (20.00)
	Epigastric pain	14 (93.33)
	Others symptoms#	5 (33.33)

* congestive heart failure, ischemic heart disease, and venous thrombosis;

† hypothyroidism and nontoxic multinodular goiter;

‡ gastritis, gastric ulcer, hernias, liver cirrhosis, hepatitis B and C, cholecystolithiasis;

§ recurrent bladder cancer and benign prostatic hyperplasia;

// tension headache, amenorrhea, hirsutism, asthma, cataracts, seizures, Chagas disease, dyslipidemia;

¶ tubal ligation, Billroth II partial gastrectomy;

# diarrhea, flushing, dysphagia and arthralgia.

The time of symptoms onset ranged from 0 to 60 months, with a mean of ten months. The most common reported symptom was epigastric pain (93.33%), followed by bloating (46.67%), and weight loss (46.67%).

The macroscopic characteristics visualized in the upper digestive endoscopy (UDE) are summarized in Table 2. In this cohort of patients, there was the predominance of 0 to 1 lesion (60%), sizing ≥1.5 cm (40%), located in the gastric antrum (53.33 %), with ulceration (60%), and Borrmann III (33.33%) classification. In addition to lesions, other conditions included pangastritis (13.33%), severe gastritis (6.67%), and stenosing pyloric ulcer (6.67%).

**Table 2:** Macroscopic findings of the upper digestive endoscopy of the 15 patients studied.

Number of lesions		n (%)
	0 a 1	9 (60.00)
	2 a 3	2 (13.33)
	Multiples	4 (26.67)
**Lesion size (cm)**		
	Not described	6 (40.00)
	<1,5	3 (20.00)
	≥1,5	6 (40.00)
**Location**		
	Pre-pyloric	3 (20.00)
	Pylorus	1 (6.67)
	Gastric antrum	8 (53.33)
	Gastric corpus	6 (40.00)
	Duodenal bulb	2 (13.33)
	Gastric fundus	1 (6.67)
	Esophagus	1 (6.67)
	Not applicable	1 (6.67)
**Ulceration**		
	Present	9 (60.00)
	Absent	6 (40.00)
**Borrmann classification**		
	Not described	9 (60.00)
	Borrmann II	1 (6.67)
	Borrmann III	5 (33.33)
**Other lesions**		
	Stenosing pylorus ulcer	1 (6.67)
	Pangastritis	2 (13.33)
	Severe gastritis	1 (6.67)

Surgical procedure was indicated in most cases (80%), for curative purposes. The subtotal gastrectomy with Roux-en-Y reconstruction was the technique most applied (53.33%). In two patients, reconstruction was performed through double transit in modified Rosanov technique^29^ (13,33%); one patient underwent total gastrectomy with Roux-en-Y reconstruction; one was submitted only to Roux-en-Y gastroenterostomy due to advanced tumor (6.67%), another underwent lesion resection plus peritoneal implant resection plus liver metastasis resection (6.67%), and another had subtotal esophagectomy plus partial gastrectomy and reconstruction with gastric tube and cervical esophagogastric anastomosis (6.67%). The characteristics of the 15 cases studied, surgical procedures, recurrence, and follow-up time are summarized in Table 3.

**Table 3:** The characteristics of the 15 patients studied, surgical procedures, recurrence and follow-up time.

N°	Male/female	Age (years)	Surgical procedure	Recurrence	Follow-up time (years)
1	F	67	Subtotal gastrectomy	No	18
2	F	20	Subtotal gastrectomy	No	32
3	F	67	Subtotal gastrectomy	No	8
4	F	72	Subtotal gastrectomy	No	Postoperative death
5	F	47	Subtotal gastrectomy	No	6
6	F	77	Gastroenteroanastomosis (liver metastases)	Liver metastases	2
7	F	54	Subtotal gastrectomy	Liver metastases	6
8	M	47	Suture of perforated gastric ulcer	Bone and liver metastases	2
9	F	49	Partial gastrectomy plus liver metastases exeresis	Liver metastases	12
10	F	44	Subtotal gastrectomy	No	8
11	M	60	Subtotal gastrectomy	No	11
12*	F	65	Subtotal esophagectomy plus proximal gastrectomy	No	9
13*	M	65	Subtotal gastrectomy plus liver metastases exeresis plus splenectomy	Liver metastases	2
14	F	48	Subtotal gastrectomy	Liver metastases (right hepatectomy)	1
15	M	68	Subtotal gastrectomy	Liver metastases	1

* Concomitant squamous cell carcinoma of the distal esophagus.

The histopathological findings of the biopsies performed by UDE, the histopathological results of the surgical specimens, and the immunohistochemical tests are in Table 4. No neuroendocrine differentiation was found in the biopsy obtained by UDE in eight cases (53.33%), with a predominance of gastric adenocarcinoma (46.67%). Of the total patients, seven (46.67%) were positive for chromogranin A and synaptophysin. The Ki-67 index was evaluated in seven cases, with two presenting values between 0 and 2%, four between 3 and 20%, and one >20%. On the other hand, in the analysis of surgical specimens, events of invasive neuroendocrine tumor predominated (60%), there was margin involvement in four cases (26.67%), lymph node invasion in seven (46.67%), neural invasion in six (40%), and angiolymphatic in 12 (80%).

**Table 4:** Histopathological and immunohistochemical analysis after upper digestive endoscopy and in the surgical specimens of the 15 patients studied.

Histopathological diagnosis by upper digestive endoscopy		n (%)
	Gastric adenocarcinoma	7 (46.67)
	Carcinoid tumor	2 (13.33)
	Well differentiated neuroendocrine tumor	3 (20.00)
	Moderate differentiated carcinoma with differentiation neuroendocrine	1 (6.67)
	Chronic gastritis	1 (6.67)
	Squamous cell carcinoma of distal esophagus and gastric neuroendocrine neoplasia	1 (6.67)
**Immunohistochemical analysis after upper digestive endoscopy**		**n (%)**
	Positive chromogranin A	7 (46.67)
	Positive synaptophysin	7 (46.67)
	Ki-67: 0 to 2%	2 (13.33)
	Ki-67: 3 to 20%	4 (26.67)
	Ki-67: >20%	1 (6.67)
	Ki-67 not performed	8 (53.33)
**Histopathological diagnosis by surgical specimen**		**n (%)**
	Gastric neuroendocrine tumor associated to gastric adenocarcinoma	5 (33.33)
	Invasive gastric neuroendocrine tumor	9 (60.00)
	Infiltrative neuroendocrine tumor	1 (6.67)
	Margin involvement	4 (26.67)
	Lymph node metastases	7 (46.67)
	Neural invasion	6 (40.00)
	Angiolymphatic invasion	12 (80.00)
**Histopathological diagnosis by surgical specimen**		**n (%)**
	Positive chromogranin A	9 (60.00)
	Positive synaptophysin	10 (66.67)
	Ki-67: 0 to 2%	5 (33.33)
	Ki-67: 3 to 20%	2 (13.33)
	Ki-67: >20%	4 (26.67)
	Ki-67 not performed	4 (26.67)

The immunohistochemical analysis recorded positive chromogranin A in nine patients (60%), and positive synaptophysin in ten (66.67%). The Ki-67 study was carried out in 11 cases showing a rate between 0 and 2%, two cases between 3 and 20%, and four >20%.

Postoperative hospitalization time ranged from 8 to 30 days, with an average of 14 days. Nine patients had postoperative complications, and the surgical wound infections were the most common (26.67%). One patient had severe pulmonary complications and died (6.67%).

The follow-up time ranged from 1 to 32 years, with a mean of 7.87 years. In the latest follow-up, tomography was performed in 13 cases (86.67%), with liver metastases observed in five patients (33.33%), lymph nodes in two (13.33%), bone metastases in one (6.67%), and peritoneal carcinomatosis in one (6.67%). Octreoscan was performed in five cases (33.33%), showing liver and bone metastases and peritoneal carcinomatosis in one of them (6.67%). During the follow-up period, three patients (20.00%) had tumor recurrence and five (33.33%) were reoperated. Adjuvant treatment with octreotide was performed in two patients (13.33%), chemotherapy in four (26.67%), and combined chemotherapy and radiotherapy in four (26.67%).

## DISCUSSION

NETs are rare neoplasms, representing about 2% of gastric tumors^17,37^. The incidence is higher in female patients, over 60 years of age, as observed in this study, due to the hormonal profile^13^, the higher prevalence of atrophic gastritis in women, or associated genetic factors^22^.

The evaluation of comorbidities is essential in the therapeutic decision. In a study by Darbà and Marsà^8^ in 2019, arterial hypertension was the main comorbidity presented by patients, corroborating this study. This finding is possibly due to the late age of presentation of both diseases^8^.

The smoking and alcoholism are important risk factors for several neoplasms; their impact on gastric NETs is still uncertain, with weak association or absence^7^.

Gastric NETs present slow growth and are usually non-functional, differing from other gastrointestinal tumors^15^. Associated clinical manifestations include abdominal pain, anemia, upper gastrointestinal bleeding, and weight loss. However, a significant number of cases may be asymptomatic^5^. Epigastric pain was the most frequent clinical manifestation, reinforcing the importance of considering this diagnosis in the investigation of symptoms.

The UDE is essential in the diagnosis of gastric NETs, and its findings are variable. Type I and II tumors usually present as multiple nodules in the gastric fundus and body, smaller than 1 to 2 cm. Type III tumors are single lesions, generally larger than 2 cm, located mainly in the gastric antrum and fundus. And type IV tumors generally manifest as multiple small lesions^5,20,31^. In this study, there was a predominance of cases with single lesions, larger than 1.5 cm, and located in the gastric antrum – a profile compatible with type III.

Biopsies and histopathological investigation are also essential for treatment. It is recommended that, in addition to the biopsy of the lesion, a biopsy of the surrounding mucosa should be performed to identify atrophic gastritis, intestinal metaplasia, and hyperplasia^15^. Besides, the immunohistochemical study of chromogranin A and synaptophysin are indispensable markers, considering that the histopathological evaluation can be nonspecific and that false-negative results may occur, with diagnostic doubt about other histological types^5^.

CT and magnetic resonance imaging (MRI) are sufficient for staging in most cases^3^. Scintigraphy with radioactive octreotide (linked to indium-111), also known as Octreoscan, can help expose primary tumors or metastases not detected in conventional imaging tests^3^.

The Octreoscan was not performed in all cases (only in four in the preoperative period and in five in the follow-up). In the preoperative, bone metastasis was identified in one patient and lymph node metastases in another, which were not diagnosed by CT.

Clinical, radiological, as well as histological staging are of great importance in analyzing tumor behavior and determining surgical therapy. The histological grade can be defined by the mitotic rate and/or Ki-67 index, classifying NETs into low-grade tumors (G1), with a Ki-67 index from 0 to 2%; intermediate grade (G2), with a Ki-67 index from 3 to 20%; and high-grade tumors (G3), with a Ki-67 index >20%^16,24^.

In eight cases in this study, the initial diagnosis was gastric adenocarcinoma, without previous evidence of NET. However, in the histopathological evaluation of the surgical specimen, the immunohistochemical examination showed the NETs, predominantly G2 tumors. The difference obtained between the evaluation of the biopsy by UDE and the surgical specimen can be explained by intratumoral heterogeneity, which justifies the need to evaluate more mitotically active areas to minimize evaluation divergences^24^.

Surgery remains the best option for the treatment of gastric NETs^16^. Initially, the therapeutic definition should consider the classification proposed by Rindi et al.^34^, which determines the occurrence of three types of gastric NETs: Type I tumors, with multiple lesions smaller than 1 cm, well-differentiated histology, hypergastrinemia, and association with atrophic gastritis and anemia pernicious; Type II tumors, with clinical and laboratory features similar to type I tumors, differing by their association with Zollinger-Ellison syndrome and multiple endocrine neoplasia type I; Type III tumors, characterized by a single ulcerated lesion and histology ranging from well-differentiated to moderately differentiated; and Type IV tumors, characterized by single and large lesions, poorly differentiated and with a high infiltrative and metastatic potential^26,34^.

Types I and II tumors should be evaluated by size, number of lesions, invasion of the muscularis propria layer, and lymph node metastasis. Tumors smaller than 1 cm, with up to five lesions and without muscle or lymph node invasion can be observed or submitted to endoscopic resection. Surgical treatment is the best option when the invasion of the muscularis propria or serosa is diagnosed, and gastrectomy with lymph node resection is recommended^34^. Gastrectomy with lymphadenectomy is indicated for tumors between 1 and 2 cm; for type I and II tumors larger than 2 cm, with six or more lesions, and with muscle layer invasion or lymph node metastasis; and for types III and IV tumors^12,16,38^.

The surgical technique employed may include local excision with endoscopic resection, local resection, subtotal gastrectomy, and total gastrectomy, among others^11,12,25^.

Type I tumors have low risk of metastases, so conservative treatment is recommended especially in smaller lesions, where endoscopic mucosal resection or endoscopic submucosal dissection should be applied. Antrectomy may be an option, especially in cases of hypergastrinemia or recurrence. Finally, somatostatin analogues, such as octreotide, may be a therapeutic option in inoperable cases or in cases of metastatic disease^12,25^.

In type II tumors, resection of the gastrinoma is the surgical approach of choice, and antrectomy does not have a favorable effect^1^. In types III and IV tumors, the preferred treatment is subtotal or total gastrectomy with D2 lymphadenectomy, as in gastric adenocarcinomas^12,34^. Resectable liver metastases must be surgically treated. Arterial embolization, radioablation, and liver transplantation are indicated when tumors are unresectable. Chemotherapy should be considered in cases of extrahepatic metastases or recurrent symptomatic disease^35,36^. In this study, cases treated with subtotal gastrectomy and Roux-en-Y reconstruction with lymphadenectomy predominated. This profile is mainly due to the definition of a cohort of patients who required surgical treatment and the surgical protocols adopted.

Previous studies showed that patients undergoing a gastrectomy presented pulmonary, anastomotic, and cardiac complications^18^. In this study, only two patients had pulmonary complications and three had anastomotic, predominantly surgical wound infections. Prophylactic measures are important, such as nursing care and antibiotic therapy, as well as new surgical approaches, as minimally invasive surgery, which reduces surgical wound infections and pain^18^.

The follow-up of patients must be individualized, according to tumor type and local guidelines. The recommendation of the National Comprehensive Cancer Network is a clinical history, physical examination, UDE, CT or MRI of the abdomen, and serum chromogranin A every six months, for one to two years, followed by once a year for four years, and biennially up to ten years after surgery^23,36^.

Finally, considering tumor recurrence, we observed, in this study, that 20% of cases had recurrence during follow-up and 33.3% required some reoperation, either due to recurrence or management of complications. Data regarding the recurrence of gastric NETs remain heterogeneous. Studies with type I tumors indicated recurrence in 63.6% and 52% of cases^9^. Lin et al.^27^ observed tumor recurrence in 47.5% of cases of gastric neuroendocrine carcinoma^27^. In events of recurrence after endoscopic resections, gastrectomy is indicated, in addition to other therapies such as chemotherapy, radiotherapy, and somatostatin analogues^2,6^.

## CONCLUSIONS

Gastric NETs have a low incidence in the general population. However, their study gains importance in view of the specificities of each case and the need for adequate management to prevent recurrences and complications. Although endoscopic treatment and minimally invasive surgery have become relevant in the management of these tumors, conventional surgery should still be considered in a significant number of cases, especially in the presence of type III and IV tumors.

## References

[1] Barchi LC, Ramos MFKP, Dias AR, Forones NM, Carvalho MP, Castro OAP (2021). Brazilian gastric cancer association guidelines (part 2): update on treatment. Arq Bras Cir Dig.

[2] Basuroy R, Srirajaskanthan R, Prachalias A, Quaglia A, Ramage JK (2014). Review article: the investigation and management of gastric neuroendocrine tumours. Aliment Pharmacol Ther.

[3] Bombardieri E, Ambrosini V, Aktolun C, Baum RP, Bishof-Delaloye A, Del Vecchio S (2010). 111In-pentetreotide scintigraphy: procedure guidelines for tumour imaging. Eur J Nucl Med Mol Imaging.

[4] Cao LL, Lu J, Lin JX, Zheng CH, Li P, Xie JW (2018). Incidence and survival trends for gastric neuroendocrine neoplasms: an analysis of 3523 patients in the SEER database. Eur J Surg Oncol.

[5] Corey B, Chen H (2017). Neuroendocrine tumors of the stomach. Surg Clin North Am.

[6] Crosby DA, Donohoe CL, Fitzgerald L, Muldoon C, Hayes B, O’Toole D (2012). Gastric neuroendocrine tumours. Dig Surg.

[7] Curtin K, Cannon-Albright LA, VanDerslice J, Yu Z, Herget KA, Thota R (2019). Associations of tobacco and alcohol use with risk of neuroendocrine tumors of the small intestine in Utah. Cancer Epidemiol Biomarkers Prev.

[8] Darbà J, Marsà A (2019). Exploring the current status of neuroendocrine tumours: a population-based analysis of epidemiology, management and use of resources. BMC Cancer.

[9] Daskalakis K, Tsoli M, Karapanagioti A, Chrysochoou M, Thomas D, Sougioultzis S (2019). Recurrence and metastatic potential in type 1 gastric neuroendocrine neoplasms. Clin Endocrinol (Oxf).

[10] Debelenko LV, Emmert-Buck MR, Zhuang Z, Epshteyn E, Moskaluk CA, Jensen RT (1997). The multiple endocrine neoplasia type I gene locus is involved in the pathogenesis of type II gastric carcinoids. Gastroenterology.

[11] Fave GD, Kwekkeboom DJ, Van Cutsem E, Rindi G, Kos-Kudla B, Knigge U (2012). ENETS Consensus guidelines for the management of patients with gastroduodenal neoplasms. Neuroendocrinology.

[12] Fave GD, O’Toole D, Sundin A, Taal B, Ferolla P, Ramage JK (2016). ENETS Consensus Guidelines update for gastroduodenal neuroendocrine neoplasms. Neuroendocrinology.

[13] Dias AR, Azevedo BC, Alban LBV, Yagi OK, Ramos MFKP, Jacob CE (2017). Gastric neuroendocrine tumor: review and update. Arq Bras Cir Dig.

[14] Dobru D, Boeriu A, Mocan S, Pascarenco O, Boeriu C, Molnar C (2013). Gastric carcinoids and therapeutic options. Case report and review of the literature. J Gastrointestin Liver Dis.

[15] Dromain C, Baere T, Lumbroso J, Caillet H, Laplanche A, Boige V (2005). Detection of liver metastases from endocrine tumors: a prospective comparison of somatostatin receptor scintigraphy, computed tomography, and magnetic resonance imaging. J Clin Oncol.

[16] Eto K, Yoshida N, Iwagami S, Iwatsuki M, Baba H (2020). Surgical treatment for gastrointestinal neuroendocrine tumors. Ann Gastroenterol Surg.

[17] Fraenkel M, Faggiano A, Valk GD (2015). Epidemiology of neuroendocrine tumors. Front Horm Res.

[18] Francischetto T, Pinheiro VPSF, Viana EF, Moraes ED, Protásio BM, Lessa MAO (2023). Early postoperative outcomes of the esophagectomy minimally invasive in esophageal cancer. Arq Bras Cir Dig.

[19] Gladdy RA, Strong VE, Coit D, Allen PJ, Gerdes H, Shia J (2009). Defining surgical indications for type I gastric carcinoid tumor. Ann Surg Oncol.

[20] Gluckman CR, Metz DC (2019). Gastric neuroendocrine tumors (carcinoids). Curr Gastroenterol Rep.

[21] Hirabayashi K, Zamboni G, Nishi T, Tanaka A, Kajiwara H, Nakamura N (2013). Histopathology of gastrointestinal neuroendocrine neoplasms. Front Oncol.

[22] Kaizaki Y, Fujii T, Kawai T, Saito K, Kurihara K, Fukayama M (1997). Gastric neuroendocrine carcinoma associated with chronic atrophic gastritis type A. J Gastroenterol.

[23] Kargwal N, Panda V, Jha A, Singh CB (2021). Gastric neuroendocrine tumor. Surg J (N Y).

[24] Klimstra DS, Modlin IR, Coppola D, Lloyd RV, Suster S (2010). The pathologic classification of neuroendocrine tumors: a review of nomenclature, grading, and staging systems. Pancreas.

[25] Kunz PL, Reidy-Lagunes D, Anthony LB, Bertino EM, Brendtro K, Chan JA (2013). Consensus guidelines for the management and treatment of neuroendocrine tumors. Pancreas.

[26] Li TT, Qiu F, Qian ZR, Wan J, Qi XK, Wu BY (2014). Classification, clinicopathologic features and treatment of gastric neuroendocrine tumors. World J Gastroenterol.

[27] Lin J, Zhao Y, Zhou Y, Tian Y, He Q, Lin J (2021). Comparison of survival and patterns of recurrence in gastric neuroendocrine carcinoma, mixed adenoneuroendocrine carcinoma, and adenocarcinoma. JAMA Netw Open.

[28] Lloyd RV, Osamura RY, Klöppel G, Rosai J (2017). WHO classification of tumours of endocrine organs.

[29] Lopes LR, Cesconetto DM, Coelho JS, Andreollo NA (2011). The modified Rosanov technique in the reconstruction of digestive tract after total gastrectomy. ABCD Arq Bras Cir Dig.

[30] Niederle MB, Hackl M, Kaserer K, Niederle B (2010). Gastroenteropancreatic neuroendocrine tumours: the current incidence and staging based on the WHO and European Neuroendocrine Tumour Society classification: an analysis based on prospectively collected parameters. Endocr Relat Cancer.

[31] Nikou GC, Angelopoulos TP (2012). Current concepts on gastric carcinoid tumors. Gastroenterol Res Pract.

[32] Patel N, Barbieri A, Gibson J (2019). Neuroendocrine tumors of the gastrointestinal tract and pancreas. Surg Pathol Clin.

[33] Rindi G, Bordi C, Rappel S, La Rosa S, Stolte M, Solcia E (1996). Gastric carcinoids and neuroendocrine carcinomas: pathogenesis, pathology, and behavior. World J Surg.

[34] Rindi G, Luinetti O, Cornaggia M, Capella C, Solcia E (1993). Three subtypes of gastric argyrophil carcinoid and the gastric neuroendocrine carcinoma: a clinicopathologic study. Gastroenterology.

[35] Sampaio RL, Coelho GR, Quidute ARP, Rocha DR, Soares CEL, Garcia JHP (2023). Liver transplant for metastatic neuroendocrine tumors: a single-center report. Arq Bras Cir Dig.

[36] Silveira F, Basile ML, Kuga FS, Próspero JD, Paes RAP, Bernardi FDC (2017). Neuroendocrine tumors: an epidemiological study of 250 cases at a tertiary hospital. Rev Assoc Med Bras (1992).

[37] Taal BG, Visser O (2004). Epidemiology of neuroendocrine tumours. Neuroendocrinology.

[38] Wang R, Zheng-Pywell R, Chen HA, Bibb JA, Chen H, Rose JB (2019). Management of gastrointestinal neuroendocrine tumors. Clin Med Insights Endocrinol Diabetes.

[39] Yang Z, Tang LH, Klimstra DS (2011). Effect of tumor heterogeneity on the assessment of Ki67 labeling index in well-differentiated neuroendocrine tumors metastatic to the liver: implications for prognostic stratification. Am J Surg Pathol.

